# Prevalence, virulence profiles and antibiotic susceptibility patterns of Shiga toxin producing *Escherichia coli* O157:H7 among children 6–59 months in Longido, Arusha-Tanzania

**DOI:** 10.1371/journal.pone.0353396

**Published:** 2026-07-10

**Authors:** Martin Michael Martin, Haikael David Martin, Beatus Modesty Lyimo

**Affiliations:** 1 Department of Food Biotechnology and Nutritional Sciences, School of Life Sciences and Bioengineering, the Nelson Mandela African Institution of Science and Technology (NM-AIST), Arusha, Tanzania; 2 Department of Molecular Biology and Biotechnology (MBB), College of Natural and Applied Sciences (CoNAS), University of Dar es Salaam (UDSM), Dar es Salaam, Tanzania; 3 Department of Health and Bio-Medical Sciences, School of Life Sciences and Bioengineering, the Nelson Mandela African Institution of Science and Technology (NM-AIST), Arusha, Tanzania; CINVESTAV: Centro de Investigacion y de Estudios Avanzados del Instituto Politecnico Nacional, MEXICO

## Abstract

Shiga toxin producing *Escherichia coli* (STEC) is a zoonotic pathogen associated with diarhoeal disease and severe complications in children, yet its epidemiology in pastoral settings of Tanzania remains insufficiently characterized. This study determined the prevalence, virulence gene profiles and antibiotic susceptibility patterns of STEC among children aged 6‍-59 months with diarhoea in Longido District, northern Tanzania. A hospital based cross-sectional‍ study was conducted between July and August 2025, enrolling 150 participants from four health facilities. Stool samples were collected and analyzed using culture, serological conformation and multiplex polymerase chain reaction targeting five genes; *rfbE*, *stx1*, *stx2*, *eaeA* and *hlyA*. STEC was operationally defined by detection of *stx1* and/or *stx2*. Antibiotic susceptibility was assessed using the Kirby-Bauer disk diffusion method. The prevalence of STEC was‍ 13.3% (20/‍150). All *stx2* positive isolates co-occurred with *stx1*. Virulence genes showed a heterogeneous but significantly clustered distribution, with *rfbE* (20.0%) and stx1 (13.3%) predominating. Significant co-occurrence was observed‍ between *stx1* and *eae* and between *stx1* and *hlyA* (p < .001). Animal contact, raw milk consumption and use of untreated water were significantly associated with STEC infection. Firth penalized logistic regression confirmed these exposures as independent predictors. Antibiotic susceptibility profiles were uniform, with complete susceptibility to ciprofloxacin,‍ gentamicin, cefotaxime and ceftazidime while ampicillin and trimethoprim showed complete resistance. These findings indicate that STEC transmission in pastoral communities is strongly driven by zoonotic and environmental exposures, characterized by clustered virulence determinants and consistent antibiotic profiles. Limitations include the cross-sectional design and short sampling period, which may not capture seasonal variation. Strengthened surveillance and integrated One Health interventions are needed to reduce disease burden.

## Introduction

Shiga toxin producing *Escherichia coli* (STEC), particularly the O157:H7 serotype, is an important zoonotic pathogen responsible for food and water-borne diarrhoeal disease globally [1]. Transmission occurs primarily through contaminated food or water and direct or indirect contact with animal reservoirs, especially ruminants, which constitute the principal natural host [2]. In humans, STEC‍ infection ranges from mild, self-limiting diarrhoea to severe complications such as hemorrhagic colitis and hemolytic uremic syndrome (HUS) [3]. The HU‍S is a life threatening condition characterized by acute renal failure, hemolytic anaemia and thrombocytopenia, with the highest risk in young children and other vulnerable population [4]. Disease severity is largely mediated by Shiga toxins (Stx1 and Stx2), which damage endothelial cells and disrupt vascular integrity [5].

S‍TEC is a recognized cause of global foodborne outbreaks linked to contaminated meat, dairy products, fresh produce and water sources [6]. It is estimated to cause 2.8 million acute illness annually, resulting in considerable health care costs [7]. Despite this burden, the epidemiology of STEC remains insufficiently characterized in many low and middle-income countries [7]. Limited laboratory capacity and weak surveillance systems contribute to underdiagnosis and underreporting, obscuring the true disease burden [8]. In Sub Sahara Africa, diarrhoeal diseases remain a leading cause of morbidity and mortality among children under five years of age [9]. Diarrhoeagenic *Escherichia coli‍* is a major cause of diarrhoeal disease, but data on specific pathotypes like STEC remain limited. Prevalence estimates vary widely due to differences in study design, diagnostic methods and limited molecular techniques, underscoring the need for standardized epidemiological studies.

In Tanzania, diarrhoeal disease continues to pose a major public health challenge in young children [10,11]. *Escherichia coli* is a leading etiological agent, and S‍TEC has increasingly been recognized as an important zoonotic contributor. Earlier studies reported relatively low prevalence of‍ STEC among diarrhoeagenic *E. coli* isolates including O157:H7 [12]. More recent studies, however, suggest higher detection rates, likely reflecting improved molecular diagnostic methods such as polymerase chain reaction (PCR) [13]. These findings indicate that the burden of STEC‍ may previously have been underestimated. The epidemiology‍ of STEC in Tanzania is likely influenced by environmental, behavioral and socioeconomic factors, particularly‍ in pastoral communities [12]. In districts such as Longido, close human livestock interaction, consumption of unpasteurized milk, undercooked animal products and limited access to safe water and sanitation create favorable conditions for zoonotic transmission of enteric pathogens [14]. This highlight the importance of a One Health approach integrating human, animal and environmental health.

Antimicrobial resistance (AMR) further complicates the management of‍ enteric infections. The AMR, defined as the ability of microorganisms to withstand antimicrobial agents, is a growing global health threat [15]. In low and middle-income countries, widespread and often unregulated use of antibiotics in humans and livestock promotes selection and dissemination of resistance organisms [15]. Studies in East Africa have reported high resistance to commonly used antibiotics, including co-trimoxazole, ampicillin and gentamicin, among enteric bacterial‍ pathogens [13]. In Tanzania, multidrug-resistant and extended spectrum beta-lactamase (ESBL) producing Enterobacteriaceae have also been reported,‍ underscoring the need for continuous surveillance.

Despite increasing recognition of STEC as a public health concern, comprehensive data on its prevalence, virulence gene profiles and antimicrobial susceptibility patterns remain limited, particularly in pastoral settings where transmission dynamics may differ from urban areas. This gap restricts evidence based prevention and control strategies and limits the ability of health systems to design targeted interventions, strengthen surveillance and improve clinical management of diarrhoeal diseases in high-risk‍ populations. Generating such evidence is essential for informing One Health based approaches, guiding antimicrobial stewardship and supporting policy decisions aimed at reducing the burden of enteric infections among vulnerable groups. Therefore, this study aimed to determine the prevalence of STEC, characterize its virulence determinants and assess antimicrobial susceptibility patterns among children aged 6‍-59 months‍ presenting with diarhoea in Longido District.

## Methodology

### Study design

This was a hospital based cross-sectional study conducted between July and August 20‍25. Data were collected at a single point in time from each participant, enabling the assessment of variables of interest and the determination of the prevalence, virulence profiles and antimicrobial susceptibility patterns of STEC in the target population.

### Settings

The research was done in four health facilities in Longido District, Arusha, Tanzania. The health facilities included in the research were Eworendeke health center, Namanga dispensary, Engarenaibor health center and Longido District hospital.

The facilities were selected based on the fact that they cover a large population from different areas and essentially cover the entire Longido District.

### Participants/samples

The study employed a convenience sampling technique to recruit children aged 6–59 months presenting with diarrhoea at health facilities in Longido District during the study period [16]. This method was suitable for recruiting human subject at the hospital based settings as it enabled the inclusion of consecutively available eligible participants, ensuring efficient case enrolment within the limited timeframe. Socio demographic and exposure data, including age, sex, clinical symptoms, water source, animal contact and dietary practices, were collected using a structured caregiver questionnaire. Clinical classification of diarrhoeal cases followed the WH‍O Integrated Management of Childhood Illness (IMCI) guidelines to ensure standardization. Children with chronic conditions such as sickle cell anaemia‍ and tuberculosis were excluded from the study.

### Microbiology, serology and molecular methods

Approximately 150 fecal samples were collected from children using sterile, dry, leak-proof, wide-mouthed plastic containers (Neomedic Limited,‍ China). Samples were maintained at 4 °C in cool boxes with ice packs and transported to the NM-AI‍ST laboratory for analysis [17]. Approximately 10g of each sample was pre-enr iched in 90 mL‍ of modified trypton soy broth (mTS B; Oxoi d Ltd., UK) and incubated at 41 °C for 24 h [18].Enriched samples were streaked onto Cefixime Tellurite Sorbitol MackConkey agar (CT-S‍MAC; Oxoid Ltd., UK) and incubated at 37 °C for 24 h [19]. Non- sorbitol fermenting colonies were considered presumptive STEC O1‍57:H7 and subcultured onto Eosin Methylene Blue agar (HiMedia Laboratories, India) for further characterization [19].

Serological confirmation was performed using an *E. coli* O15 7 latex agglutination test (Oxoid Ltd.) [20]. Genomic DNA was extracted using the QIAamp PowerFecal Pro DNA Kit (Qiagen) [21]. Multiplex PCR was employed to simultaneously detect five key virulence-associated genes of *Escherichia coli* O157:H7: *rfbE*, *stx*1, *stx*2, *eaeA* and *hlyA*. The *rfbE* gene was used to confirm the O15‍7 serogroup, while *stx1* and *stx2* encode Shiga toxins 1 and 2, the principal toxins responsible for STEC pathogenicity. The *eaeA* gene encodes intimin, an adhesion protein that mediates attachment of the bacterium to intestinal epithelial cells, whereas *hlyA* encodes enterohemolysin, a virulence factor associated with host cell damage. Amplification of these target genes was performed using a Bio-Rad Laboratories C1000 Touch Thermal Cycler. The‍ primer sequences used for each target gene are listed in Table 1, and the composition of the PCR master mix is provided in Table 2. [22].

**Table 1:** Primer used in detection of STEC species specific gene and virulent genes.

Primer	Direction	Primer sequence (5′-3′)	Size (bases)	Reference
*rfbE*	Forward Reverse	AAGATTGCGCTGAAGCCTTTG CATTGGCATCGTGTGGACAG	497	[20]
*EHEC* *hly*	Forward Reverse	ACGATGTGGTTTATTCTGGA CTTCACGTGACCATACATAT	165	[23]
*stx1*	Forward Reverse	ACACTGGATGATCTCAGTGG CTGAATCCCCCTCCATTATG	614	[24]
*stx2*	Forward Reverse	CCATGACAACGGACAGCAGTT CCTGTCAACTGAGCAGCACTTTG	779	[24]
*eaeA*	Forward Reverse	GTGGCGAATACTGGCGAGACT CCCCATTCTTTTTCACCGTCG	890	[24]

*Note*; Primer sequences and amplicon sizes from previously published studies [20,23,24].

**Table 2:** Master mix solution for *rfbE*, *Stx1*, *Stx2*, *eaeA* and *hlyA* genes.

Reagents	1x Reaction Volume (µL)
*rfbE* forward primer	0.5
*rfbE* reverse primer	0.5
*EHEC-*hly forward primer	0.5
*EHEC-hly* reverse primer	0.5
*stx1 forward primer*	0.5
*stx1 reverse primer*	0.5
*stx2 forward primer*	0.5
*stx2 reverse primer*	0.5
*eaeA forward primer*	0.5
*eaeA reverse primer*	0.5
2x Multiplex mix	6.25
Deionized water	0.75
Template DNA	3
**Total**	**15**

*Note*; The multiplex PCR master mix composition for target genes amplification [20,24,25].

The thermo cycler was programmed with standard parameters to perform the PCR reactions. The parameters used were: an initial denaturation step, followed by 35 cycles of amplification and finally a step for the last extension [26].The reaction mixture was first denatured at 95 °C for 15 minutes, followed by 35 cycles of amplification. Each cycle consisted of denaturation at 95 °C for 30 seconds, annealing at 55 °C for 90 seconds, and final extension at 72 °C for 90 seconds. After the amplification, 10 µL of the PCR products was analyzed on a 1.5% agarose gel in 5x Tris-borate-EDTA buffer, with the DNA staining solution containing 0.1 µL/mL GelRed (Phenix Research) [27]. The electrophoresis was done at 100 V for 1 hour, with a 1000 bp PCR ladder used as the size marker. The gel was then photographed under a UV transilluminator with a digital camera [28].

### Antibiotic susceptibility test

The susceptibility of the positive STEC isolates to various‍ antibiotics was tested using the Kirby-Bauer disk diffusion technique [29]. According to the guidelines of the Clinical Laboratory Standards Institute, six antibiotics commonly used in the treatment of bacterial infections in Arusha, Tanzania, were chosen [29]. The antibiotics used in the study were: Ciprofloxacin (CIP5),‍ Gentamicin (G EN10), Cefotaxime (CTX3‍0), Trimethoprim (COT25), Cefta zidime (CAZ30) and Ampic illin (AMP10) (Termo Fisher Scientific-Oxoid, Basingstoke, Hampshire, UK). The reference for the Kirby- Bauer disk diffusion assay was a McFarland standard of 0.5, which is equivalent to 10^8 CFU‍/ml [30]. The process started with the bacteria saline mixture being poured onto a 15 x 150 mm Mueller-Hinton agar plate, which was left to set for‍ five minutes. The antibiotic disc were then placed on the plates and the plates were incubated at 37 °C for 24 hours [31]. After the incubation period, the zone of inhibition was measured with a transparent ruler to determine the‍ Kirby-Bauer disk‍ diffusion results [32]. The assessment of the bacteria’s response to the antibiotic was done‍ using the reference standard, which was STEC O157: H7, ATCC 43895, which had never been exposed to antibiotics before [32]. The results obtained were interpreted using the chart of the Clinical Laboratory Standards Institute (January 2025) and the isolates were classified as multidrug resistant (MDR) when they exhibited resistance to at least one antimicrobial agent in three or more antimicrobial classes [33].

### Statistical analysis

Data were entered, cleaned and prepared using Microsoft Excel. Descriptive statistics were used to summarize socio-demo graphic and health related characteristics, and were presented as frequencies, percentages, means and standard deviations. The prevalence of STEC positive samples was calculated using CDC Epi Info ™ version 7.2.7 [34].

Associations between categorical variables were assessed using Pearson’s chi-‍square test or Fisher’s exact test, as appropriate [34]. All estimates were reported with 95% confidence intervals and statistical significance was defined at a p-value < 0.05. All further analyses were performed using IBM SPSS statistics version 29 (IBM Corp., Armonk, NY, USA). Variables with p < 0.20 in bivariate‍ analysis were included in multivariable logistic regression models. Binary logistic regression was initially used to identify factors associated with Shiga toxin one *stx1* gene detection. Due to sparse data and quasi-complete separation, Firth’s penalized logistic regression was applied to obtain bias reduced estimates [35]. Adjusted odds ratios (AORs) and corresponding 95% confidence intervals‍ (95% CIs) were reported.

### Ethical clearance

Ethical approval was obtained from the National Institute for Medical Research Health Research Ethics Committee, Tanzania (Ref. No. N IMR/HQ/R.8a/Vol. IX/4867). Written informed consent was obtained from all participants. Data were anonymized, stored securely and used solely for research‍ purposes to ensure confidentiality and to protect participants’ privacy throughout.

## Results

### Distribution of exposure related practices, environmental factors and reported clinical signs among study participants

As shown in Table 3, most study participants reported frequent exposure to potential risk factors associated with STEC infections. Animal exposure was reported by 103 participants (68.‍7%‍), while 77 participants (51.3%) reported consumption o f undercooked beef. A high proportion of respondents reported intake of unpasteurized milk (81.3‍%) and use of untreated water sources (83.3%). Regarding clinical‍ manifestation diarrhoea was the most commonly reported signs, affecting 88 participants (5 8.7%), followed by fever in 53 participants (35.3%‍) and body weakness in 9 participants (6.0%) (Table 3).

**Table 3:** Distribution of selected exposure -related practices, environmental factors and reported clinical signs among study participants (N = 150).

Variable	Category	n	%
Animal exposure	Yes	103	68.7
	No	47	31.3
Consuption of undercooked beef	Yes	77	51.3
	No	73	48.7
Raw milk intake	Yes	122	81.3
	No	28	18.7
Water source	Untreated	125	83.3
	Treated	25	16.7
Clinical signs	Diarrhoea	88	58.7
	Fever	53	35.3
	Body weakness	9	6.0

*Note*; Percentages were calculated using the total sample size (N = 150). Reported clinical signs were not mutually exclusive; therefore, participants could report more than one symptom.

### Prevalence of STEC and associated virulence genes

A s shown in Table 4, the overall prevalence of STEC was 13.3% (20/15 0), defined by the detection of *stx1*. All *stx2*-positive isolates were concurrently positive for *stx1*, indicating co-occurrence of these virulence genes. Virulence genes showed a heterogeneous distribution, with statistically significant difference in observed frequencies (all p < 0.001). The most frequently detected gene was *rfbE* (20.0%), followed by‍ *stx1* (13.3%). Lower frequencies were observed for *stx2* and *eaeA* (8.0‍% each) and *hlyA* (7.3%). The 95% confidence intervals (Table 4) were narrower for higher frequency genes (*rfbE* and *stx1*) compared with lower-frequency genes. Chi-square goodness of fit tests indicated significant differences‍ between observed and expected distributions f or all genes (χ² =  54.00–109. 36, df = 1, p < 0.001). S1 Fig. shows representative agarose gel electrophoresis of PCR amplicons from selected STEC-positive isolates, confirming detection of virulence genes in representative samples (N011B, N003BZL, and N021). The gel pro vides qualitative confirmation of PCR detection and is consistent with the prevalence data presented in Table‍ 4.

**Table 4:** Prevalence of STEC and associated virulence genes (N = 150).

Gene	Detected n (%)	95% CI (%)	χ² (df = 1)	p-value
*stx1*	20 (13.3)	[7.9, 18.7]	80.67	<.001
*stx2*	12 (8.0)	[3.7, 12.3]	105.84	<.001
*rfbE*	30 (20.0)	[13.6, 26.4]	54.00	<.001
*eaeA*	12 (8.0)	[3.7, 12.3]	105.84	<.001
*hly*	11 (7.3)	[3.2, 11.5]	109.36	<.001

*Note*; Values are expressed as n (%) with 95% CIs; χ² tests (df = 1) indicate significant deviations from expected distribution for all genes (p < .001).

### Virulence genes and STEC status in *Escherichia coli* isolates

Chi-square analysis indicated a significant association between O157:H7 coding gene *rfbE* and STEC stat us (χ² = 45.62, p < .001), with all STEC isolates linked to O157:H7 strains. Significant co-occurrence patterns were also observed between Shiga toxin one *stx1* and intimin *eaeA* (χ² = 18.74, p < .001) and between Shiga toxin one *stx1* and hemolysin gene *hlyA* (χ² = 16.21, p < .001), indicating clustering of toxin and adherence/hemolysin genes. In‍ addition, Shiga toxin two *stx2* showed a significant association with accessory virulence determinants (χ² = 9.88, p = .002). Overall, Table 5 demonstrates a nonrandom distribution of virulence genes, reflecting strong genetic linkage among pathogenic *E. coli* isolates.

**Table 5:** Associations between virulence genes in *Escherichia coli* isolates (N = 150).

Gene Association	χ² (df = 1)	p-value
*rfbE* vs STEC status	45.62	< 0.001
*stx1* vs *eaeA*	18.74	< 0.001
*stx1* vs *hly*	16.21	< 0.001
*stx2* vs accessory virulence genes	9.88	0.002

*Note*; Associations were assessed using Pearson’s chi-square test (df = 1). STEC was defined as the presence of stx1 and/or stx2. All tests were performed at a significance level of p < 0.05.

### Exposure factors and *stx1* gene detection among participants

As shown in “Table 6”, bivariate analysis identified significant association between Shiga toxin one *stx1* detection and selected exposure variables. Animal exposure was significantly associated with positivity (χ² = 10.764, p = 0. 005), with all positive cases occurring among exposed individuals. Similarly, raw milk consumption showed a significant association (χ² = 5.2 96, p = 0.021), with *stx1* detection restricted to consumers‍. In contrast, undercooked beef consumption demonstrated a non-significant association (χ² = 3.219, p = 0.073), and water source was not significantly associated with *stx1* status (χ² = 2.262, p = 0.133). Overall, “Table 6” indicates that zoonotic exposure pathways particularly animal contact and raw milk intake, were the main determinants of STEC detection in the study population.

**Table 6:** Association between exposure factors and *stx1* gene detection (N = 150).

Variable	Category	*stx1* detected n (%)	*stx1* not detected n (%)	χ² (df)	p-value
Animal exposure	Yes	18 (18.9)	77 (81.1)	10.764 (2)	0.005*
	No livestock	0 (0.0)	47 (100.0)	–	–
	Other “YES” group	2 (25.0)	6 (75.0)	–	–
Undercooked beef consumption	Yes	14 (18.2)	63 (81.8)	3.219 (1)	0.073
	No	6 (8.2)	67 (91.8)	–	–
Raw milk intake	Yes	20 (16.4)	102 (83.6)	5.296 (1)	0.021*
	No	0 (0.0)	28 (100.0)	–	–
Water source	Untreated	19 (15.2)	106 (84.8)	2.262 (1)	0.133
	Treated	1 (4.0)	24 (96.0)	–	–

*Note*; Values are presented as n (%) and analyzed using Pearson’s chi-square test (df as indicated), with p < .05 indicating statistical significance; “-”reference categories not independently tested.

### Firth penalized logistic regression of factors associated with *stx1* gene detection

Firth penalized logistic regression was applied to correct for quasi complete separation and small sample bias, as shown in Table 7. After adjustment, animal exposure remained significantly associated with Shiga toxin one *stx1* detection (AOR = 2‍.1, 95% CI: 1.1–4.3, p = 0.028). Similarly, raw milk consumption showed a strong positive association (AOR = 3.4, 95% CI: 1.5–7.9, p = 0.003). Use of untreated water was also significantly associated with increased odds of *stx‍1* positivity (AOR = 2.7, 95% CI: 1.1–6.8, p = 0.031). In contrast, undercooked beef consumption was not significantly related to the outcome (AO‍R = 1.2, 95% CI: 0.6–2.5, p = 0.610). Overall, Table 7 indicates that zoonotic and environmental exposures, particularly animal contact, raw milk intake and the use of untreated water, were independent predictors of STEC infection through *stx1* gene detection after bias correction.

**Table 7:** Firth penalized logistic regression of factors associated with *stx1* gene (N = 150).

Variable	Category	AOR	95% CI	p-value
Animal exposure	Yes vs No	2.1	1.1–4.3	0.028
Raw milk intake	Yes vs No	3.4	1.5–7.9	0.003
Undercooked beef	Yes vs No	1.2	0.6–2.5	0.610
Untreated water	Yes vs Treated	2.7	1.1–6.8	0.031

*Note*; Adjusted odds ratios (AOR) with 95% confidence intervals (CI) were obtained from a Firth penalized logistic regression model, with p < .05 considered statistically significant.

### Antibiotic susceptibility test of STEC positive isolates

Antibiotic susceptibility testing of the 20 STEC-positive isolates showed a dichotomous resistance pattern as presented in Table 8. All isolate s‍ (100%) were susceptible to ciprofloxacin, gentamicin, cefotaxime and ceftazidime, while all isolates (100%) were resistant to trimethoprim and ampicillin. No intermediate susceptibility was observed for any of the antibiotics tested. Zone diameters were interpreted according to‍ the Clinical and Laboratory Standards Institute (CLSI) Performance Standards for Antimicrobial Susceptibility Testing (M100, 35^th^ ed., 2025), a s summarized in S1 Appendix. Overall, Table 8 shows uniform susceptibility to ciprofloxacin, gentamicin and third generation cephalosporin and complete resistance‍ to ampicillin and trimethoprim among the isolates.

**Table 8:** Antibiotic susceptibility profile of STEC positive isolates (N = 20).

Antibiotic	Susceptible n (%)	Resistant n (%)
Ciprofloxacin (CIP)	20 (100.0)	0 (0.0)
Gentamicin (GEN)	20 (100.0)	0 (0.0)
Cefotaxime (CTX)	20 (100.0)	0 (0.0)
Ceftazidime (CAZ)	20 (100.0)	0 (0.0)
Trimethoprim (COT)	0 (0.0)	20 (100.0)
Ampicillin (AMP)	0 (0.0)	20 (100.0)

*Note*; Values are presented as frequencies (n) and percentages (%).

### Distribution of virulence genes and antimicrobial susceptibility patterns among isolates

As shown in Table 9, all isolates (N = 2‍0) displayed uniform antimicrobial‍ susceptibility profiles characterized by complete susceptibility (100.0%) to Ciprofloxacin, Gentamicin, Cefotaxime and Ceftazidime, alongside complete resistance (100.‍0%) to Trimethoprim and Ampicillin. I n contrast, virulence gene distribution showed variability across isolates, with *stx1* and *rfbE* detected in all isolates (100.0%), while‍ *stx2* and *eaeA* were present in 60.0% each, and *hlyA* in 55.0%. Despite heterogeneity in virulence gene‍ prevalence, antibiotic susceptibility patterns remained invariant across all isolates. Owing to the absence of variability in susceptibility outcomes, inferential statistic testing of association between virulence determinants and resistance phenotypes was not feasible, and analyses were limited to descriptive summaries.

**Table 9:** Distribution of virulence genes and antimicrobial susceptibility patterns (N = 20).

Gene	Detected n (%)	Not detected n (%)	Antibiotic susceptibility pattern
*stx1*	20 (100.0)	0 (0.0)	All susceptible to CIP, GEN, CTX, CAZ; all resistant to COT and AMP
*rfbE*	20 (100.0)	0 (0.0)	
*stx2*	12 (60.0)	8 (40.0)	
EHEC *hlyA*	11 (55.0)	9 (45.0)	
*eaeA*	12 (60.0)	8 (40.0)	

*Note*; Values are presented as frequencies (n) and percentages (%).

## Discussion

This study examined the epidemiology, virulence characteristics and antibiotic susceptibility patterns of Shiga toxin producing *Escherichia coli* (O157:H7) among children with diarrhoeal disease in a pastoral setting in northern Tanzania. The study revealed moderate STEC prevalence (13.3%), a heterogeneous yet‍ significantly clustered distribution of virulence genes dominated by *rfbE* and *stx‍1*, and a uniform antibiotic susceptibility profile. Zoonotic and environmental exposures, particularly animal contact, consumption of unpasteurized milk and the use of untreated water emerged as key determinants of STEC infection in the study population.‍

The observed prevalence aligns with recent studies from Sub-Saharan Africa reporting STEC rates between 10% and 20% among children with diarrhoea [36,37] Comparable findings have been reported in East African pastoral and agro-pastoral communities, where close human animal contacts facilitate transmission. Earlier report in Tanzania revealed lower prevalence [12], whereas more recent studies indicate increasing detection, likely due to improved molecular diagnostics‍ [13]. The higher prevalence in this study may therefore reflect both methodological sensitivity and the high-risk exposure context of Longido District.

The predominance of *rfbE* and its strong association with STEC status is consistent with regional evidence identifying O157:H7 as a dominant zoonotic strain [38,39]. The observed co-occurrence of *stx1*, *eaeA*, and *EHEC hlyA* further supports findings from Uganda and Kenya, where virulence gene clustering enhances bacterial pathogenesis [20,40]. In contrast, the relatively lower prevalence of *stx2* differs from Southern African studies linking this gene to more severe disease outcomes [41], suggesting geographic variation in circulating strains and virulence profiles.

The‍ significant associations between exposure factors and STEC detection reinforce the importance of zoonotic and environmental transmission pathways. Animal contact and unpasteurized milk consumption were strongly associated with infection, consistent with studies in Tanzania and neighboring countries [42,43]. These findings reflect the role of livestock as reservoirs and highlight risks associated with traditional practices such as consumption of unpasteurized milk. The association with untreated water further indicates environmental contamination as a contributing factor. Although undercooked beef consumption was not statistically significant, it remains an established risk factor and may be influenced by contextual dietary practice or sample size limitations. Biologically, the clustering of virulence genes suggest the circulation of genetically related strains with coordinated expression of toxin, adhesion, and hemolysin factors, which may enhance colonization and disease severity [44]. Methodologically, the us e of multiplex PCR improved detection sensitivity, while Firth penalized logistic regression strengthened inference by addressing small‍-sample bias.

The antibiotic susceptibility findings revealed a uniform patterns, with complete susceptibility to ciprofloxacin, gentamicin, cefotaxime‍, and ceftazidime and complete resistance to trimethoprim and ampicillin. This pattern is partly consistent with regional‍ studies reporting high resistance to first line antibiotics [45]. However, the absence of resistance to higher generation antibiotics contrasts with emerging resistance trends reported elsewhere [39]. This uniformity may reflect limited selective pressure or clonal circulation of strains within the study population.

The public health implications are considerable. The strong link between STEC infection and zoonotic exposure underscores the need for integrated one health approaches, including improved livestock hygiene, safe milk processing and access to clean water. In addition antimicrobial stewardship programs are essential to preserve the effectiveness of currently active drugs. Strengthened surveillance systems incorporating molecular techniques are critical for monitoring pathogen dynamics and resistance trends.‍

Several limitations should be considered. First, samples were collected over a short period (July–August‍ 2025) and seasonal variation in enteric pathogens may influence prevalence estimates; thus‍, longitudinal studies across different seasons are needed to provide a more comprehensive epidemiological picture. Second, the lack of variability in antibiotic susceptibility outcomes limited the ability to assess statistical associations between resistance patterns and virulence genes. However, this uniformity may reflect consistent resistance profiles, potentially influenced by shared selective pressures or clonal relatedness, although this was not directly assessed. Additionally, the cross-sectional design limits causal inference and the relatively small number of STEC positive isolates may have reduced statistical power.

In conclusion, STEC infections in this setting are shaped by zoonotic and environmental exposures‍ and characterized‍ by distinct virulence gene patterns and consistent antibiotic susceptibility profiles. These findings highlight the importance of targeted interventions at the human animal environment interface. Future research should incorporate longitudinal and genomic approaches to better understand seasonal dynamics, transmission pathways, and the evolution of virulence genes and resistance patterns.

## Supporting information

S1 Fig Representative Agarose gel‍ electrophoresis of PCR amplicons demonstrating detection of virulence associated genes in Shiga toxin producing *Escherichia coli* (STEC) isolates. 1 kb ladder = 1000 bp molecular size marker; (−VE) = negative control; (+VE‍) = positive control (*E. coli* O 157:H7 A TCC‍ 43895). Representative STEC positive isolates shown in number 7, 11 and 15 correspond to sample identifiers N011B, N003BZL, and N021, respectively. Expected amplicon sizes were *eaeA* (890 bp), *stx2* (779 bp), *s‍tx1* (6‍14 bp), *rfbE* (497 bp) and *hly A* (165 bp). The image is representative of multiple independent PCR assays; therefore, not all isolates analyzed in this study are displayed the same gel. Brightness and contrast were adjusted uniformly across the entire image solely to enhance visualization of D‍NA bands, without selective modification of any region‍.
(TIF)

S1 Raw Image Original, uncropped gel corresponding to S1 Fig. (JPG)

S1 Appendix Antibiotic zone diameter interpretive chart.‍ Interpretive criteria for antimicrobial susceptibility testing based on‍ inhibition zone diameters (mm) for ciprofloxacin (CIP5), gentamicin (GEN10), cefotaxime (CTX30), trimethoprim (COT25)‍, ceftazidime (CAZ30), and ampicillin (AMP10). Classification of susceptible, intermediate and resistant isolates was performed according to Clinical and Laboratory Standards Institute (CLSI) guidelines. Source: CLSI. Performance Standards for Antimicrobial Susceptibility Testing. 3 5^th^ ed. CLSI supplement M10 0. Clinical and Laboratory Standards Institute; 2025.
(PDF)

S1 Dataset The STEC prevalence data. Dataset containing participants-level STEC⁠ prevalence data and associated variables used in the statistical analyses reported in this study.
(XLSX)

S2 Dataset STEC virulence genes and antibiotic susceptibility profiles. Dataset containing STEC virulence gene profiles and antibiotic susceptibility data used in the analyses reported in this study.
(XLSX)
