# Supplementary figures and images for: Prevalence, virulence profiles and antibiotic susceptibility patterns of Shiga toxin producing Escherichia coli O157:H7 among children 6–59 months in Longido, Arusha-Tanzania
Source: PLoS One. 2026 Jul 10;21(7):e0353396. doi: 10.1371/journal.pone.0353396 (PMC13353950; doi:10.1371/journal.pone.0353396)

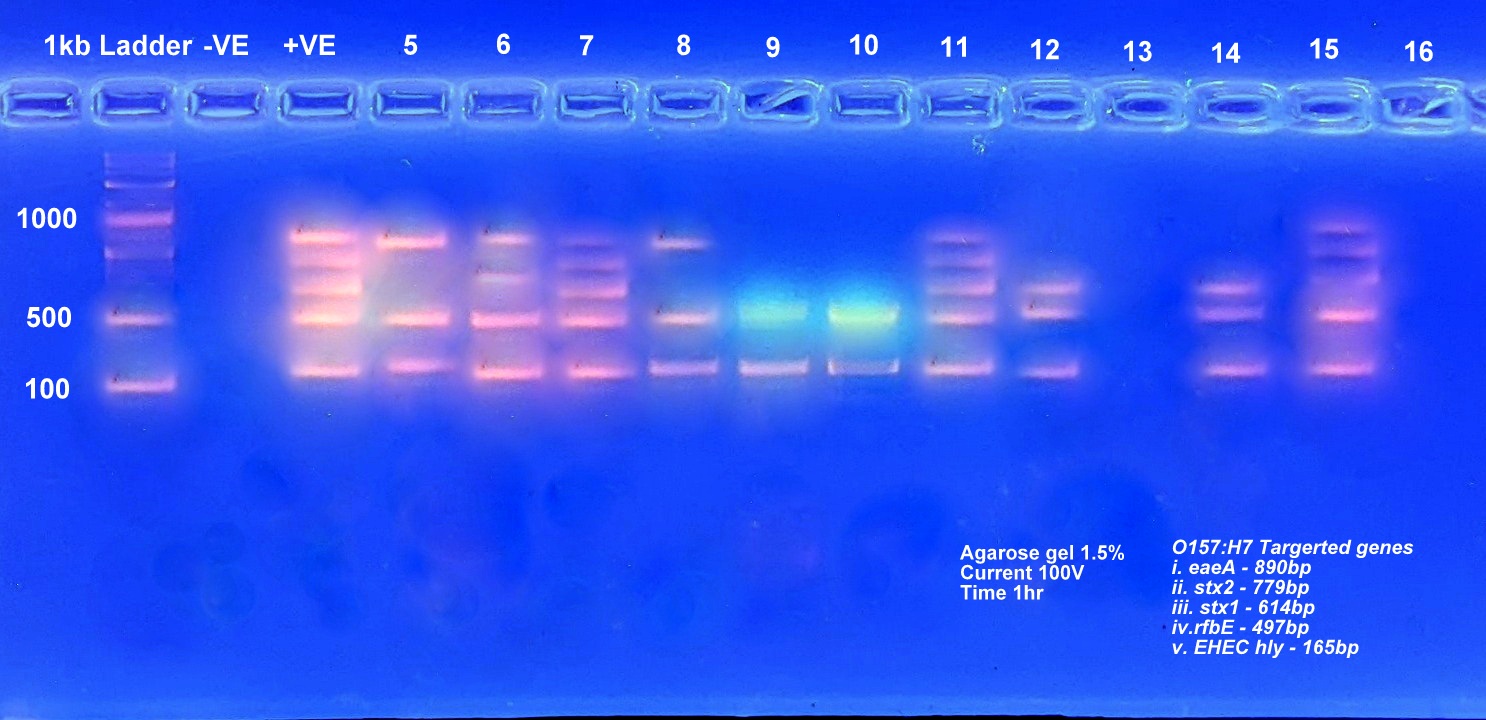

Supplement: S1 Raw Image — (JPG) [file pone.0353396.s002.jpg]
