# Supplementary material for: Prevalence, virulence profiles and antibiotic susceptibility patterns of Shiga toxin producing Escherichia coli O157:H7 among children 6–59 months in Longido, Arusha-Tanzania
Source: PLoS One. 2026 Jul 10;21(7):e0353396. doi: 10.1371/journal.pone.0353396 (PMC13353950; doi:10.1371/journal.pone.0353396)
Supplement: S1 Appendix — Interpretive criteria for antimicrobial susceptibility testing based on‍ inhibition zone diameters (mm) for ciprofloxacin (CIP5), gentamicin (GEN10), cefotaxime (CTX30), trimethoprim (COT25)‍, ceftazidime (CAZ30), and ampicillin (AMP10). Classification of susceptible, intermediate and resistant isolates was performed according to Clinical and Laboratory Standards Institute (CLSI) guidelines. Source: CLSI. Performance Standards for Antimicrobial Susceptibility Testing. 3 5th ed. CLSI supplement M10 0. Clinical and Laboratory Standards Institute; 2025. (PDF) [file pone.0353396.s003.pdf]

S1 Appendix 1: Antibiotic zone diameter interpretive chart

Antibiotic (Disk Content)	Inhibition zone (mm)		
	Susceptible	Intermediate	Resistance
Ciprofloxacin (CIP5)	>=26	22-25	<=21
Gentamicin (GEN10)	>=18	15-17	<=14
Cefotaxime (CTX30)	>=26	23-25	<=22
Trimethoprim (COT25)	≥ 16	11–15	≤ 10
Ceftazidime (CAZ30)	>=21	18-20	<=17
Ampicillin (AMP10)	>=17	14-16	<=13

Source: CLSI. Performance Standards for Antimicrobial Susceptibility Testing. 35th ed. CLSI supplement M100. Clinical and Laboratory Standards Institute; 2025.
