# Supplementary material for: Prevalence, virulence profiles and antibiotic susceptibility patterns of Shiga toxin producing Escherichia coli O157:H7 among children 6–59 months in Longido, Arusha-Tanzania
Source: PLoS One. 2026 Jul 10;21(7):e0353396. doi: 10.1371/journal.pone.0353396 (PMC13353950; doi:10.1371/journal.pone.0353396)
Supplement: Attachment — Submitted filename: Letter_response_to_reviewers.pdf [file pone.0353396.s008.pdf]

THE UNITED REPUBLIC OF TANZANIA
MINISTRY OF EDUCATION, SCIENCE, AND TECHNOLOGY
THE NELSON MANDELA
AFRICAN INSTITUTION OF SCIENCE AND TECHNOLOGY (NM-AIST)

SCHOOL OF LIFE SCIENCES AND BIOENGINEERING (LiSBE)

In reply please quote:
Ref. No: NM-AIST/P048/T23 **Date:** 07th May, 2026

PLOS ONE

Dear Editors and Reviewers,

RE: Response to reviewers and submission of revised manuscript for PLOS ONE

We sincerely thank you for the time and effort invested in reviewing our manuscript and for the constructive comments provided. We have carefully addressed all the concerns raised by the reviewers and have revised the manuscript accordingly. In particular, we have strengthened the methodological rigor and data analysis by incorporating additional variables,including human-animal interaction, dietary patterns and access to treated or untreated water, as key exposure factors associated with Shiga toxin Producing *E. coli* infection. These variables were part of the original data collection but were inadvertently omitted from the initial STEC prevalence dataset, as they were retained for another objective within the same study. The data have now been retrieved, verified for accuracy and incorporated into the analysis. All statistical analyses have been re-run, including Firth logistic regression, to ensure robustness and consistency of the findings. We have also improved the clarity and structure of the manuscript by refining the introduction, enhancing the results with appropriate statistical analyses, and strengthening the discussion to align with the findings. Issues related to microbiological characterization, terminology and data presentation has been corrected as suggested. Additionally, we have clarified the definition and classification of STEC based on virulence genes, revised prevalence estimates accordingly, improved figures and tables, and addressed all minor corrections noted by the reviewers. A detailed, point by point response to each reviewer comment is provided in the accompanying table.

We believe that these revisions have significantly improved the quality, clarity and scientific rigor of the manuscript.

Thank you for your consideration.

Sincerely,

M.Michael

Mr. Martin Michael Martin (Corresponding author)

Academia for Society and Industry
Tengeru Campus, Nelson Mandela Road, P.O Box 447, Tengeru, Arusha; Mobile: +255762596915, Email: martinm@nm-aist.ac.tz; Website: www.nm-aist.ac.tz

TABLE X, MATRIX OF REVIEWERS' COMMENTS AND AUTHORS' RESPONSES

REVIEWER	REVIEWERS COMMENT	HOW IT WAS ADDRESSED	SECTION
Reviewer 1	The samples were collected between July and August 2025. Considering the potential seasonal variation of enteric pathogens, could the authors discuss whether the reported prevalence may vary across other seasons?	○ Thank you for this comment. We acknowledge that sampling was conducted between July and August 2025, and seasonal variation may influence enteric pathogen virulence.<br>○ Therefore the reported prevalence may not reflect other seasons.<br>○ This limitation has been noted in the revised manuscript, and we recommend future longitudinal studies to address seasonal variation.	Methodology
	The discussion attributes the high prevalence to factors such as human–animal interaction, sanitation practices and food habits; however, these variables were not directly measured in the study. Could the authors clarify whether these interpretations are supported by collected data or moderate this conclusion?	○ Thank you for insightful comment. Proposed explanatory factors has been revisited.<br>○ Relevant data on exposure variables related to human – animal interaction, access to safe water and food habits were re-visited and re-examined and their association with STEC prevalence was determined through bivariate analysis.<br>○ These results have now been incorporated into the manuscript to support interpretation. Discussion section has been revised to align with the analyzed data.	Results and discussion
	The definition of Shiga toxin producing *Escherichia coli* O157:H7 relies on the presence of stx1 and/or stx2 genes. However, several isolates (N014, N069, N045, N050, N079, N115, N032, N010B, N006B, N014B) reported in Table "ANTIBITIC_SUSCEPTITY_DATA_PLOS _ONE_SUBMISSION" appear to be positive for the rfbE gene but negative for both *stx1* and *stx2*. Could the authors clarify whether these isolates were still classified as STEC and how this was considered in the prevalence calculation?	○ Thank you for this comment, we have clarified and revised the manuscript accordingly<br>○ Specifically, the prevalence of STEC was re-evaluated based on the presence of *stx1* and/or *stx2* genes and now is reported as 13.3%, while the prevalence of *E. coli* O157:H7 serotypes remains 20%<br>○ The antibiotic susceptibility data were reanalyzed based on 13% confirmed STEC isolates	Results
	The table "ANTIBITIC_SUSCEPTITY_DATA_PLOS _ONE_SUBMISSION" includes both virulence gene detection and antimicrobial susceptibility data. For clarity, the authors may consider either revising the table title to reflect both datasets or separating the information into two tables.	○ The table title has been revised accordingly to clearly reflect the association between virulence genes detection and antimicrobial susceptibility patterns in STEC positive isolates	Results
	In addition, this dataset is currently provided in the supplementary material. It would improve readability if a summarized table of virulence gene prevalence and antimicrobial susceptibility were included in the main manuscript.	○ A summarized table (Table 3) of virulence gene prevalence and antimicrobial susceptibility patterns has been included in the revised manuscript	Results
	In Figure 2, the correspondence between gel lanes and the Sample IDs listed in Table "ANTIBITIC_SUSCEPTITY_DATA_PLOS _ONE_SUBMISSION" is not indicated. The authors should clarify which isolates correspond to each lane.	○ Gel wells were labeled numerically due to limited space on the gel doc.<br>○ The figure just provided a representative image from multiple PCR runs, so not all isolates appeared in the same gel.<br>○ The correspondence between lane numbers and sample IDs (e.g., lane 7 = N011B, lane	Results

		11 = N003BZL, lane 15 = N021) has now been clarified in the figure legend.	
	In addition, the expected band sizes for each virulence gene should be annotated in the gel, and the image quality (resolution/contrast) could be improved to facilitate interpretation of the PCR products.	○ Thank you for this valuable comment<br>○ The expected band sizes for each virulence gene have now been clearly indicated in the figure and described in the legend to facilitate interpretation<br>○ While we acknowledge the limitation in image resolution due to the original gel documentation, we have optimized the contrast as much as possible to improve visibility.	Results
	Remove STEC in the tittle<br>Line 68 = change "Shiga toxin-producing E. coli STEC" by only "STEC"<br>Line 239 = change "aea" to "eae"<br>Line 323 = change "Shiga toxin producing *E.coli*" to "STEC"<br>Line 334 = change "aea" to "eae"<br>Line 334 = "…hemolysin gene EHEC hly was identified…" this gene is the hlyA? describe the full name of the hemolysin gene	○ All terminology has been standardized by replacing redundant phrase with "STEC" and correcting typographical errors (e.g., "aea" to "eae").<br>○ The hemolysin gene has been clarified as hlyA and fully described as enterohemorrhagic *Escherichia coli* hemolysin A (EHEC hlyA).<br>○ All changes have been incorporated into the revised manuscript.	Tittle page, Introduction and Methodology
REVIEWER #2	While the study is interesting and the frequency of STEC O157:H7 (20%) is striking, the article requires a more in-depth analysis of the collected data. A more thorough analysis of the sociodemographic and clinical information in relation to the isolates obtained is lacking. In all cases, only percentage values are mentioned, without any correlation analysis. It is noteworthy that, given the high frequency of STEC, these patients were not followed up. Another weakness of the manuscript is the presentation of the results, which presents raw data without adequate statistical analysis. The manuscript provides too much information, not necessarily related, especially in the introduction. The discussion is weak, and given the lack of statistical analysis, it is not possible to draw clear conclusions.	○ In-depth statistical analysis has been conducted using Firth logistic regression.<br>○ Correlation between STEC prevalence and exposure factors (human–animal contact, dietary habits, and access to clean and safe water) has been analyzed and included.<br>○ Results section has been improved to include statistical outputs beyond percentages.<br>○ Discussion has been strengthened to reflect analytical findings and support clearer conclusions.<br>○ Introduction has been revised to remove irrelevant information and improve focus.<br>○ Overall manuscript has been revised to enhance clarity, coherence, and scientific rigor.	Introduction, Methodology, Results and Discussion
REVIEWER #3	However, there are several methodological, conceptual, and structural limitations that need to be addressed. These include issues related to study design, microbiological characterization, and data presentation. In addition, several aspects of the manuscript would benefit from improved clarity, updated references, and better organization. In its current form, the manuscript requires major revision before it can be considered for publication.	○ We thank the reviewer for the comments.<br>○ All concerns have been addressed, and the manuscript has been revised to improve methodology, data presentation, clarity, organization and references.	Introduction, Methodology, Results and Discussion
