# Supplementary material for: Prevalence, virulence profiles and antibiotic susceptibility patterns of Shiga toxin producing Escherichia coli O157:H7 among children 6–59 months in Longido, Arusha-Tanzania
Source: PLoS One. 2026 Jul 10;21(7):e0353396. doi: 10.1371/journal.pone.0353396 (PMC13353950; doi:10.1371/journal.pone.0353396)
Supplement: Attachment — Submitted filename: Response to Reviewers.pdf [file pone.0353396.s009.pdf]

THE UNITED REPUBLIC OF TANZANIA
MINISTRY OF EDUCATION, SCIENCE, AND TECHNOLOGY
THE NELSON MANDELA
AFRICAN INSTITUTION OF SCIENCE AND
TECHNOLOGY (NM-AIST)

SCHOOL OF LIFE SCIENCES AND BIOENGINEERING (LiSBE)

In reply please quote:
Ref. No: NM-AIST/P048/T23 **Date:** 17th June, 2026

PLOS ONE

Dear editors and reviewers,

Re; Letter of response to reviewers comments

Refer to heading above,

We sincerely thank you for the time and effort invested in evaluating our manuscript and for the constructive and insightful comments provided. We have carefully addressed all concerns raised and revised the manuscript accordingly. In response to the reviewer's comments, we updated the epidemiological information by replacing older estimates with more recent data and references from 2020. We also revised the statement regarding the global burden of Shiga toxin-producing *Escherichia coli* (STEC) to provide a specific quantitative estimate, indicating that STEC is responsible for approximately 2.8 million acute illnesses annually. To improve clarity and readability, the manuscript has undergone thorough language editing and proofreading. Repetitive wording, duplicate phrases, grammatical errors, typographical mistakes and punctuation inconsistencies identified by the reviewer have been corrected throughout the manuscript. Table citations and formatting have also been revised to comply with journal requirements. We further clarified the definition of multidrug resistance (MDR) by explicitly defining it as resistance to at least one antimicrobial agent in three or more antimicrobial classes according to internationally accepted criteria. This definition has been added to the Methods section. In addition, all discrepancies between the tracked changes and clean manuscript versions, including the ethical clearance section, have been resolved to ensure consistency. A detailed point by point response to reviewers comment is provided in the accompanying response table. Thank you for your consideration.

Sincerely,

M.Michael

Mr. Martin Michael Martin (Corresponding author)

TABLE X, MATRIX OF REVIEWERS' COMMENTS AND AUTHORS' RESPONSES

REVIEWER	REVIEWERS COMMENT	HOW IT WAS ADDRESSED	SECTION
Reviewer # 3	The statement "It is estimated to cause millions of infections annually, with a proportion progressing to severe disease requiring hospitalization" lacks quantitative support. How many millions of infections are being referred to?	○ Thank you for this comment. We have revised the statement to provide a specific quantitative estimate. The text now reads: "It is estimated to cause 2.8 million acute illnesses annually. The corresponding reference has been retained to support this estimate.	Introduction
	The authors should provide up-to-date epidemiological estimates and appropriate references to support this statement.	○ Thank you for this valuable comment. We have updated the epidemiological information by replacing the older estimates with recent data of 2020.	Introduction
	Several paragraphs still require language editing, and some grammatical and stylistic issues remain. For example, in the paragraph spanning lines 79–86, the term "Tanzania" is repeated three times unnecessarily. Additionally, in line 137, there is a repeated phrase "The results of this study provided The findings of this study"	○ Thank you for your careful review and constructive comments. The unnecessary repetition of the term " Tanzania" in lines 79–86 has been corrected and the duplicated phrase in line 137 has been removed.	Introduction
	The authors have still not clarified the criteria used to define multidrug resistance (MDR). Specifically, it remains unclear whether MDR was defined as resistance to more than two antimicrobial agents, resistance to three or more antimicrobial classes, or according to another established criterion. A clear definition should be provided.	○ Thank you for this important comment. We have clarified the definition of multidrug resistance (MDR) in the revised manuscript. MDR is now explicitly defined as acquired resistance to at least one antimicrobial agent in three or more antimicrobial classes, in line with standard international criteria.	Methodology
	The tracked-changes version also contains several typographical and grammatical errors. For example:<br>• Line 187: "Table one"<br>• Line 188: "Table two"	○ These has been revised according to the journal style.	Methodology
	In line 202, a punctuation error is present: "Bauer disk diffusion technique (Sangeda *et al.*, 2021) According to the guidelines..." A period is missing after the citation. I recommend that the authors carefully proofread the entire manuscript to identify and correct similar issues.	○ Thank you for identifying this error. We have corrected the punctuation by inserting a full stop after the citation, so that the sentence now reads appropriately.	Methodology
	I also note that the revised manuscript includes an Ethical Clearance section, whereas this information is absent from the tracked-changes version. This discrepancy further highlights the inconsistency between the two submitted documents.<br>Results	○ Thank you for this observation. We acknowledge the inconsistency between the previously submitted tracked-changes and clean version. The tracked-changes manuscript has now been updated to fully match the revised clean manuscript. Both documents have been carefully checked to ensure consistency and completeness.	Methodology
	In line 218, the authors refer to "STEC status." What do they mean by STEC Status?	○ Thank you for this comment. We acknowledge that the term "STEC status" was unclear in the original manuscript. This has been corrected in the revised version by using a clear and more precise description	Methodology & Results
