# Supplementary material for: Prevalence, virulence profiles and antibiotic susceptibility patterns of Shiga toxin producing Escherichia coli O157:H7 among children 6–59 months in Longido, Arusha-Tanzania
Source: PLoS One. 2026 Jul 10;21(7):e0353396. doi: 10.1371/journal.pone.0353396 (PMC13353950; doi:10.1371/journal.pone.0353396)
Supplement: Attachment — Submitted filename: comments.docx [file pone.0353396.s006.docx]

**General comment**

This manuscript addresses an important public health issue by investigating the prevalence, virulence gene profiles, and antimicrobial susceptibility of *Escherichia coli* O157:H7 (STEC) among children in a semi-pastoral community in Tanzania. The study is relevant within a One Health framework, particularly given the close interaction between humans, livestock, and environmental exposures in the study setting. The reported prevalence of STEC O157:H7 (20%) is striking and, if robust, represents a potentially significant contribution to the epidemiology of diarrhoeal diseases in underrepresented populations.

However, there are several methodological, conceptual, and structural limitations that need to be addressed. These include issues related to study design, microbiological characterization, and data presentation. In addition, several aspects of the manuscript would benefit from improved clarity, updated references, and better organization. In its current form, the manuscript requires major revision before it can be considered for publication.

**Introduction**

The introduction provides a general overview of STEC as a zoonotic pathogen and highlights its relevance in diarrhoeal disease. However, the logical flow of ideas needs to be improved, as the current structure is somewhat fragmented and occasionally repetitive. Some statements require careful revision for accuracy. For example, the assertion that “the majority of diarrhoeal disease outbreaks, sporadic cases, and severe clinical diseases such as hemorrhagic colitis and hemolytic uremic syndrome are attributed to STEC infection” (Lines 46 – 48) is an overstatement and not fully supported by current epidemiological evidence. While STEC is indeed an important pathogen, it is not responsible for the majority of diarrhoeal disease cases globally.

The introduction would also benefit from the inclusion of more recent references. Epidemiological data on STEC prior to 2020 are now considered outdated in many contexts, and more recent studies should be incorporated to reflect current trends. That said, it is understood that in the African context, data on STEC are limited, and the use of older studies may sometimes be unavoidable. This limitation should be acknowledged explicitly.

Additionally, the objective of the study should be reformulated to avoid implying causality. The current phrasing suggests that the study aims to “reveal the roles” of STEC in diarrhoeal disease, which is not appropriate for a cross-sectional design. A more accurate formulation would focus on determining prevalence and molecular characteristics.

**Methodology**

The methodology section describes a hospital-based cross-sectional study, which is suitable for estimating prevalence but not for establishing causation.

An important omission in this section is the lack of explicit mention of ethical approval and informed consent within the Methods section itself. Although an ethics statement appears elsewhere in the manuscript, it should be clearly integrated into the methodology.

The microbiological and molecular methods are generally appropriate, including the use of selective culture media, serological confirmation, and multiplex PCR targeting key virulence genes.

The antimicrobial susceptibility testing follows CLSI guidelines, which is appropriate, but the limited panel of antibiotics restricts the interpretation of multidrug resistance. Moreover, the criteria used to define multidrug resistance are not specified. That is, more than 2 antibiotics or?

The statistical analysis is basic and limited to descriptive statistics and chi-square tests. No multivariate analysis is performed, and potential confounding factors are not addressed.

**Results**

The results section would benefit from improved organization. The subsections titled “Participant’s characteristics,” “Participant’s socio-demographic characteristics,” and “Participant’s clinical signs” should be merged into a single, cohesive section to improve readability and avoid redundancy.

There are also issues with table organization and consistency. The numbering of tables does not always follow the order in which they are cited in the text, and this should be corrected. Additionally, Table 3 presents data only for the children, despite being described as including parents or guardians. Table 6 appears to repeat data already presented in Table 3, and this redundancy should be addressed.

The reported prevalence of STEC O157:H7 is high and noteworthy, but its interpretation is limited by the absence of a control group and the inability to distinguish between infection and asymptomatic carriage. The manuscript would benefit from a clearer discussion of this distinction.

The virulence gene data raise some concerns. While it is known that some STEC O157:H7 strains may be *eae*-negative, such strains are relatively rare. The finding that only 40% of isolates carried the *eae* gene is unusual and warrants further clarification. The presentation of these results should be revised to improve clarity and facilitate interpretation.

**Discussion**

The discussion section addresses several relevant points but would benefit from improved structure and focus. It is recommended to remove the current subheadings to allow for a more fluid and integrated narrative.

The manuscript should emphasize the importance of studying antimicrobial resistance in STEC within the appropriate clinical context. Notably, STEC infections are generally not treated with antibiotics due to the potential risk of hemolytic uremic syndrome, and this important consideration is not discussed.

The consumption of unpasteurized milk in the study population is a highly relevant finding and represents a distinctive epidemiological feature of this community. The discussion would benefit from incorporating studies that have investigated STEC contamination in raw milk or similar settings, as well as comparisons with populations that have comparable dietary practices.

Another important aspect that is not addressed is the possibility of asymptomatic carriage of STEC in the study population. This is particularly relevant in endemic settings and should be discussed as a potential explanation for the observed prevalence.

Finally, the manuscript does not adequately address its limitations, including sampling bias and the constraints of the cross-sectional design. A dedicated limitations paragraph is strongly recommended.

**Minor comments**

There are several minor issues related to formatting and terminology that should be corrected. For example, references to tables should follow a consistent format, such as “Table 1” and “Table 2,” rather than “table one” or “table two.” This issue appears multiple times throughout the manuscript, including in lines 168, 169, and 219. Additionally, the term WAZ should be defined when first introduced, as it may not be familiar to all readers.
