# Supplementary material for: Prevalence, virulence profiles and antibiotic susceptibility patterns of Shiga toxin producing Escherichia coli O157:H7 among children 6–59 months in Longido, Arusha-Tanzania
Source: PLoS One. 2026 Jul 10;21(7):e0353396. doi: 10.1371/journal.pone.0353396 (PMC13353950; doi:10.1371/journal.pone.0353396)
Supplement: Peer review history

# Peer review: Decision Letter 0

**DOI:** 10.1371/journal.pone.0353396.r001

**Authors:** Fernando Navarro-Garcia

12 Apr 2026

PONE-D-26-07244

Prevalence, virulence profiles and antibiotic susceptibility patterns of Escherichia coli O157:H7 (STEC) among children 6-59 months in Longido, Arusha-Tanzania.

PLOS One

Dear Dr. Martin,

Thank you for submitting your manuscript to PLOS ONE. After careful consideration, we feel that it has merit but does not fully meet PLOS ONE’s publication criteria as it currently stands. Therefore, we invite you to submit a revised version of the manuscript that addresses the points raised during the review process.

It is very important that the authors make their best effort to improve the manuscript by carefully considering the comments of the three reviewers.

Please submit your revised manuscript by May 27 2026 11:59PM. If you will need more time than this to complete your revisions, please reply to this message or contact the journal office at plosone@plos.org. When you're ready to submit your revision, log on to https://www.editorialmanager.com/pone/ and select the 'Submissions Needing Revision' folder to locate your manuscript file.

Please include the following items when submitting your revised manuscript:

- A letter that responds to each point raised by the academic editor and reviewer(s). You should upload this letter as a separate file labeled 'Response to Reviewers'.
- A marked-up copy of your manuscript that highlights changes made to the original version. You should upload this as a separate file labeled 'Revised Manuscript with Track Changes'.
- An unmarked version of your revised paper without tracked changes. You should upload this as a separate file labeled 'Manuscript'.

If you would like to make changes to your financial disclosure, please include your updated statement in your cover letter. Guidelines for resubmitting your figure files are available below the reviewer comments at the end of this letter.

If applicable, we recommend that you deposit your laboratory protocols in protocols.io to enhance the reproducibility of your results. Protocols.io assigns your protocol its own identifier (DOI) so that it can be cited independently in the future. For instructions see: https://journals.plos.org/plosone/s/submission-guidelines#loc-laboratory-protocols. Additionally, PLOS ONE offers an option for publishing peer-reviewed Lab Protocol articles, which describe protocols hosted on protocols.io. Read more information on sharing protocols at https://plos.org/protocols?utm_medium=editorial-email&utm_source=authorletters&utm_campaign=protocols.

As the corresponding author, your ORCID iD is verified in the submission system and will appear in the published article. PLOS supports the use of ORCID, and we encourage all coauthors to register for an ORCID iD and use it as well. Please encourage your coauthors to verify their ORCID iD within the submission system before final acceptance, as unverified ORCID iDs will not appear in the published article. *Only*  the individual author can complete the verification step; PLOS staff *cannot*  verify ORCID iDs on behalf of authors.

We look forward to receiving your revised manuscript.

Kind regards,

Fernando Navarro-Garcia

Academic Editor

PLOS One

Journal Requirements:

When submitting your revision, we need you to address these additional requirements.

1. Please ensure that your manuscript meets PLOS ONE's style requirements, including those for file naming. The PLOS ONE style templates can be found at

https://journals.plos.org/plosone/s/file?id=wjVg/PLOSOne_formatting_sample_main_body.pdf and

https://journals.plos.org/plosone/s/file?id=ba62/PLOSOne_formatting_sample_title_authors_affiliations.pdf

2. The ethical approval number(s) listed in the manuscript and/or submission metadata does not match the approval number on the ethical approval document you provided. Please ensure that all approval numbers are correct.

3. Please include your tables as part of your main manuscript and remove the individual files. Please note that supplementary tables (should remain/ be uploaded) as separate "supporting information" files.

4. Thank you for stating the following in the Acknowledgments Section of your manuscript:

“The authors received no specific fund for this research”

We note that you have provided funding information that is not currently declared in your Funding Statement. However, funding information should not appear in the Acknowledgments section or other areas of your manuscript. We will only publish funding information present in the Funding Statement section of the online submission form.

Please remove any funding-related text from the manuscript and let us know how you would like to update your Funding Statement. Currently, your Funding Statement reads as follows:

“The author(s) received no specific funding for this work.”

Please include your amended statements within your cover letter; we will change the online submission form on your behalf.

5. PLOS ONE now requires that authors provide the original uncropped and unadjusted images underlying all blot or gel results reported in a submission’s figures or Supporting Information files. This policy and the journal’s other requirements for blot/gel reporting and figure preparation are described in detail at https://journals.plos.org/plosone/s/figures#loc-blot-and-gel-reporting-requirements and https://journals.plos.org/plosone/s/figures#loc-preparing-figures-from-image-files. When you submit your revised manuscript, please ensure that your figures adhere fully to these guidelines and provide the original underlying images for all blot or gel data reported in your submission. See the following link for instructions on providing the original image data: https://journals.plos.org/plosone/s/figures#loc-original-images-for-blots-and-gels.

In your cover letter, please note whether your blot/gel image data are in Supporting Information or posted at a public data repository, provide the repository URL if relevant, and provide specific details as to which raw blot/gel images, if any, are not available. Email us at plosone@plos.org if you have any questions.

6. Your ethics statement should only appear in the Methods section of your manuscript. If your ethics statement is written in any section besides the Methods, please move it to the Methods section and delete it from any other section. Please ensure that your ethics statement is included in your manuscript, as the ethics statement entered into the online submission form will not be published alongside your manuscript.

7. We note that Figure 1 in your submission contain map images which may be copyrighted. All PLOS content is published under the Creative Commons Attribution License (CC BY 4.0), which means that the manuscript, images, and Supporting Information files will be freely available online, and any third party is permitted to access, download, copy, distribute, and use these materials in any way, even commercially, with proper attribution. For these reasons, we cannot publish previously copyrighted maps or satellite images created using proprietary data, such as Google software (Google Maps, Street View, and Earth). For more information, see our copyright guidelines: http://journals.plos.org/plosone/s/licenses-and-copyright.

We require you to either (1) present written permission from the copyright holder to publish these figures specifically under the CC BY 4.0 license, or (2) remove the figures from your submission:

1. You may seek permission from the original copyright holder of Figure(s) [#] to publish the content specifically under the CC BY 4.0 license.

We recommend that you contact the original copyright holder with the Content Permission Form (http://journals.plos.org/plosone/s/file?id=7c09/content-permission-form.pdf) and the following text:

“I request permission for the open-access journal PLOS ONE to publish XXX under the Creative Commons Attribution License (CCAL) CC BY 4.0 (http://creativecommons.org/licenses/by/4.0/). Please be aware that this license allows unrestricted use and distribution, even commercially, by third parties. Please reply and provide explicit written permission to publish XXX under a CC BY license and complete the attached form.”

Please upload the completed Content Permission Form or other proof of granted permissions as an "Other" file with your submission.

In the figure caption of the copyrighted figure, please include the following text: “Reprinted from [ref] under a CC BY license, with permission from [name of publisher], original copyright [original copyright year].”

2. If you are unable to obtain permission from the original copyright holder to publish these figures under the CC BY 4.0 license or if the copyright holder’s requirements are incompatible with the CC BY 4.0 license, please either i) remove the figure or ii) supply a replacement figure that complies with the CC BY 4.0 license. Please check copyright information on all replacement figures and update the figure caption with source information. If applicable, please specify in the figure caption text when a figure is similar but not identical to the original image and is therefore for illustrative purposes only.

The following resources for replacing copyrighted map figures may be helpful:

USGS National Map Viewer (public domain): http://viewer.nationalmap.gov/viewer/

The Gateway to Astronaut Photography of Earth (public domain): http://eol.jsc.nasa.gov/sseop/clickmap/

Maps at the CIA (public domain): https://www.cia.gov/library/publications/the-world-factbook/index.html and https://www.cia.gov/library/publications/cia-maps-publications/index.html

NASA Earth Observatory (public domain): http://earthobservatory.nasa.gov/

Landsat: http://landsat.visibleearth.nasa.gov/

USGS EROS (Earth Resources Observatory and Science (EROS) Center) (public domain): http://eros.usgs.gov/#

Natural Earth (public domain): http://www.naturalearthdata.com/

8. Please include a separate caption for each figure in your manuscript.

9. We note that there is identifying data in the Supporting Information file “WHO-ANTHRO_DATA_PLOS_ONE_SUBMISSION.xlsx” and “STEC_PREVALENCE_DATA_PLOS_ONE_SUBMISSION.xlsx”. Due to the inclusion of these potentially identifying data, we have removed this file from your file inventory. Prior to sharing human research participant data, authors should consult with an ethics committee to ensure data are shared in accordance with participant consent and all applicable local laws.

Data sharing should never compromise participant privacy. It is therefore not appropriate to publicly share personally identifiable data on human research participants. The following are examples of data that should not be shared:

-Name, initials, physical address

-Ages more specific than whole numbers

-Internet protocol (IP) address

-Specific dates (birth dates, death dates, examination dates, etc.)

-Contact information such as phone number or email address

-Location data

-ID numbers that seem specific (long numbers, include initials, titled “Hospital ID”) rather than random (small numbers in numerical order)

Data that are not directly identifying may also be inappropriate to share, as in combination they can become identifying. For example, data collected from a small group of participants, vulnerable populations, or private groups should not be shared if they involve indirect identifiers (such as sex, ethnicity, location, etc.) that may risk the identification of study participants.

Additional guidance on preparing raw data for publication can be found in our Data Policy (https://journals.plos.org/plosone/s/data-availability#loc-human-research-participant-data-and-other-sensitive-data) and in the following article: http://www.bmj.com/content/340/bmj.c181.long.

Please remove or anonymize all personal information “Age”, ensure that the data shared are in accordance with participant consent, and re-upload a fully anonymized data set. Please note that spreadsheet columns with personal information must be removed and not hidden as all hidden columns will appear in the published file.

10. If the reviewer comments include a recommendation to cite specific previously published works, please review and evaluate these publications to determine whether they are relevant and should be cited. There is no requirement to cite these works unless the editor has indicated otherwise.

[Note: HTML markup is below. Please do not edit.]

Reviewers' comments:

Reviewer's Responses to Questions

**Comments to the Author**

1. Is the manuscript technically sound, and do the data support the conclusions?

The manuscript must describe a technically sound piece of scientific research with data that supports the conclusions. Experiments must have been conducted rigorously, with appropriate controls, replication, and sample sizes. The conclusions must be drawn appropriately based on the data presented.

Reviewer #1: Partly

Reviewer #2: No

Reviewer #3: Partly

**********

2. Has the statistical analysis been performed appropriately and rigorously?

Reviewer #1: Yes

Reviewer #2: No

Reviewer #3: Yes

**********

3. Have the authors made all data underlying the findings in their manuscript fully available?

The PLOS Data policy requires authors to make all data underlying the findings described in their manuscript fully available without restriction, with rare exception (please refer to the Data Availability Statement in the manuscript PDF file). The data should be provided as part of the manuscript or its supporting information, or deposited to a public repository. For example, in addition to summary statistics, the data points behind means, medians and variance measures should be available. If there are restrictions on publicly sharing data—e.g. participant privacy or use of data from a third party—those must be specified.

Reviewer #1: Yes

Reviewer #2: Yes

Reviewer #3: Yes

**********

4. Is the manuscript presented in an intelligible fashion and written in standard English?

PLOS ONE does not copyedit accepted manuscripts, so the language in submitted articles must be clear, correct, and unambiguous. Any typographical or grammatical errors should be corrected at revision, so please note any specific errors here.

Reviewer #1: Yes

Reviewer #2: No

Reviewer #3: No

**********

5. Review Comments to the Author

Please use the space provided to explain your answers to the questions above. You may also include additional comments for the author, including concerns about dual publication, research ethics, or publication ethics. (Please upload your review as an attachment if it exceeds 20,000 characters)

Reviewer #1: The manuscript submitted by Michael Martin et al. evaluates the prevalence of Escherichia coli producing Shiga toxin O157:H7 (STEC) in children with diarrhea in Longido District, Arusha, Tanzania. The detection of virulence genes confirms the pathogenic potential of the isolates, and the 20% prevalence suggests a relevant role in childhood diarrheal disease. In addition, resistance to some commonly used antibiotics was observed. The study provides important data for epidemiological surveillance and for preventing infections and complications such as Hemolytic Uremic Syndrome. The study addresses an important public health issue; however, several aspects of the manuscript require clarification and improvement before publication.

The samples were collected between July and August 2025. Considering the potential seasonal variation of enteric pathogens, could the authors discuss whether the reported prevalence may vary across other seasons?

The discussion attributes the high prevalence to factors such as human–animal interaction, sanitation practices and food habits; however, these variables were not directly measured in the study. Could the authors clarify whether these interpretations are supported by collected data or moderate this conclusion?

The definition of Shiga toxin–producing Escherichia coli O157:H7 relies on the presence of stx1 and/or stx2 genes. However, several isolates (N014, N069, N045, N050, N079, N115, N032, N010B, N006B, N014B) reported in Table “ANTIBITIC_SUSCEPTITY_DATA_PLOS_ONE_SUBMISSION” appear to be positive for the rfbE gene but negative for both stx1 and stx2. Could the authors clarify whether these isolates were still classified as STEC and how this was considered in the prevalence calculation?

The table “ANTIBITIC_SUSCEPTITY_DATA_PLOS_ONE_SUBMISSION” includes both virulence gene detection and antimicrobial susceptibility data. For clarity, the authors may consider either revising the table title to reflect both datasets or separating the information into two tables. In addition, this dataset is currently provided in the supplementary material. It would improve readability if a summarized table of virulence gene prevalence and antimicrobial susceptibility were included in the main manuscript.

In Figure 2, the correspondence between gel lanes and the Sample IDs listed in Table “ANTIBITIC_SUSCEPTITY_DATA_PLOS_ONE_SUBMISSION” is not indicated. The authors should clarify which isolates correspond to each lane. In addition, the expected band sizes for each virulence gene should be annotated in the gel, and the image quality (resolution/contrast) could be improved to facilitate interpretation of the PCR products.

Title: Remove “(STEC)”

Line 68 = change “Shiga toxin-producing E. coli STEC” by only “STEC”

Line 239 = change “aea” to “eae”

Line 323 = change “Shiga toxin producing E.coli” to “STEC”

Line 334 = change “aea” to “eae”

Line 334 = “…hemolysin gene EHEC hly was identified…” this gene is the hlyA? describe the full name of the hemolysin gene

Reviewer #2: The manuscript presents a study detecting STEC in children under 5 years of age in 4 locations in Longido, Arusha, Tanzania, during July and August 2025. 150 samples were analyzed, from which 30 STEC isolates were identified. The presence of some virulence genes and antibiotic susceptibility were determined in these STEC strains. Additionally, some sociodemographic and clinical variables were collected during sample collection.

While the study is interesting and the frequency of STEC O157:H7 (20%) is striking, the article requires a more in-depth analysis of the collected data. A more thorough analysis of the sociodemographic and clinical information in relation to the isolates obtained is lacking. In all cases, only percentage values are mentioned, without any correlation analysis. It is noteworthy that, given the high frequency of STEC, these patients were not followed up. Another weakness of the manuscript is the presentation of the results, which presents raw data without adequate statistical analysis.

The manuscript provides too much information, not necessarily related, especially in the introduction. The discussion is weak, and given the lack of statistical analysis, it is not possible to draw clear conclusions.

Reviewer #3: General comment

This manuscript addresses an important public health issue by investigating the prevalence, virulence gene profiles, and antimicrobial susceptibility of Escherichia coli O157:H7 (STEC) among children in a semi-pastoral community in Tanzania. The study is relevant within a One Health framework, particularly given the close interaction between humans, livestock, and environmental exposures in the study setting. The reported prevalence of STEC O157:H7 (20%) is striking and, if robust, represents a potentially significant contribution to the epidemiology of diarrhoeal diseases in underrepresented populations.

However, there are several methodological, conceptual, and structural limitations that need to be addressed. These include issues related to study design, microbiological characterization, and data presentation. In addition, several aspects of the manuscript would benefit from improved clarity, updated references, and better organization. In its current form, the manuscript requires major revision before it can be considered for publication.

Introduction

The introduction provides a general overview of STEC as a zoonotic pathogen and highlights its relevance in diarrhoeal disease. However, the logical flow of ideas needs to be improved, as the current structure is somewhat fragmented and occasionally repetitive. Some statements require careful revision for accuracy. For example, the assertion that “the majority of diarrhoeal disease outbreaks, sporadic cases, and severe clinical diseases such as hemorrhagic colitis and hemolytic uremic syndrome are attributed to STEC infection” (Lines 46 – 48) is an overstatement and not fully supported by current epidemiological evidence. While STEC is indeed an important pathogen, it is not responsible for the majority of diarrhoeal disease cases globally.

The introduction would also benefit from the inclusion of more recent references. Epidemiological data on STEC prior to 2020 are now considered outdated in many contexts, and more recent studies should be incorporated to reflect current trends. That said, it is understood that in the African context, data on STEC are limited, and the use of older studies may sometimes be unavoidable. This limitation should be acknowledged explicitly.

Additionally, the objective of the study should be reformulated to avoid implying causality. The current phrasing suggests that the study aims to “reveal the roles” of STEC in diarrhoeal disease, which is not appropriate for a cross-sectional design. A more accurate formulation would focus on determining prevalence and molecular characteristics.

Methodology

The methodology section describes a hospital-based cross-sectional study, which is suitable for estimating prevalence but not for establishing causation.

An important omission in this section is the lack of explicit mention of ethical approval and informed consent within the Methods section itself. Although an ethics statement appears elsewhere in the manuscript, it should be clearly integrated into the methodology.

The microbiological and molecular methods are generally appropriate, including the use of selective culture media, serological confirmation, and multiplex PCR targeting key virulence genes.

The antimicrobial susceptibility testing follows CLSI guidelines, which is appropriate, but the limited panel of antibiotics restricts the interpretation of multidrug resistance. Moreover, the criteria used to define multidrug resistance are not specified. That is, more than 2 antibiotics or?

The statistical analysis is basic and limited to descriptive statistics and chi-square tests. No multivariate analysis is performed, and potential confounding factors are not addressed.

Results

The results section would benefit from improved organization. The subsections titled “Participant’s characteristics,” “Participant’s socio-demographic characteristics,” and “Participant’s clinical signs” should be merged into a single, cohesive section to improve readability and avoid redundancy.

There are also issues with table organization and consistency. The numbering of tables does not always follow the order in which they are cited in the text, and this should be corrected. Additionally, Table 3 presents data only for the children, despite being described as including parents or guardians. Table 6 appears to repeat data already presented in Table 3, and this redundancy should be addressed.

The reported prevalence of STEC O157:H7 is high and noteworthy, but its interpretation is limited by the absence of a control group and the inability to distinguish between infection and asymptomatic carriage. The manuscript would benefit from a clearer discussion of this distinction.

The virulence gene data raise some concerns. While it is known that some STEC O157:H7 strains may be eae-negative, such strains are relatively rare. The finding that only 40% of isolates carried the eae gene is unusual and warrants further clarification. The presentation of these results should be revised to improve clarity and facilitate interpretation.

Discussion

The discussion section addresses several relevant points but would benefit from improved structure and focus. It is recommended to remove the current subheadings to allow for a more fluid and integrated narrative.

The manuscript should emphasize the importance of studying antimicrobial resistance in STEC within the appropriate clinical context. Notably, STEC infections are generally not treated with antibiotics due to the potential risk of hemolytic uremic syndrome, and this important consideration is not discussed.

The consumption of unpasteurized milk in the study population is a highly relevant finding and represents a distinctive epidemiological feature of this community. The discussion would benefit from incorporating studies that have investigated STEC contamination in raw milk or similar settings, as well as comparisons with populations that have comparable dietary practices.

Another important aspect that is not addressed is the possibility of asymptomatic carriage of STEC in the study population. This is particularly relevant in endemic settings and should be discussed as a potential explanation for the observed prevalence.

Finally, the manuscript does not adequately address its limitations, including sampling bias and the constraints of the cross-sectional design. A dedicated limitations paragraph is strongly recommended.

Minor comments

There are several minor issues related to formatting and terminology that should be corrected. For example, references to tables should follow a consistent format, such as “Table 1” and “Table 2,” rather than “table one” or “table two.” This issue appears multiple times throughout the manuscript, including in lines 168, 169, and 219. Additionally, the term WAZ should be defined when first introduced, as it may not be familiar to all readers.

**********

6. PLOS authors have the option to publish the peer review history of their article (what does this mean?). If published, this will include your full peer review and any attached files.

If you choose “no”, your identity will remain anonymous but your review may still be made public.

**Do you want your identity to be public for this peer review?**  For information about this choice, including consent withdrawal, please see our Privacy Policy.

Reviewer #1: No

Reviewer #2: No

Reviewer #3: No

**********

[NOTE: If reviewer comments were submitted as an attachment file, they will be attached to this email and accessible via the submission site. Please log into your account, locate the manuscript record, and check for the action link "View Attachments". If this link does not appear, there are no attachment files.]

To ensure your figures meet our technical requirements, please review our figure guidelines: https://journals.plos.org/plosone/s/figures

You may also use PLOS’s free figure tool, NAAS, to help you prepare publication quality figures: https://journals.plos.org/plosone/s/figures#loc-tools-for-figure-preparation.

NAAS will assess whether your figures meet our technical requirements by comparing each figure against our figure specifications.

Attachment Submitted filename: comments.docx

# Author comment: Author response to Decision Letter 1

**DOI:** 10.1371/journal.pone.0353396.r002

22 May 2026

COMMENTS FROM REVIEWER 1

1.The samples were collected between July and August 2025. Considering the potential seasonal variation of enteric pathogens, could the authors discuss whether the reported prevalence may vary across other seasons?

Response

(Thank you for this comment. We acknowledge that sampling was conducted between July and August 2⁠02⁠5, and seasonal variation may influence enteric pathogen virulence.Therefore the reported⁠ prevalence may not

reflect other seasons.This limitation has been noted in the revised manuscript, and we recommend future longitudinal studies to address seasonal variation).

2. The discussion attributes the high prevalence to factors such as human–animal interaction, sanitation practices and food habits; however, these variables were not directly measured in the study. Could the authors

clarify whether these interpretations are supported by collected data or moderate this conclusion?

Response

(Thank you for insightful comment. Proposed explanatory factors has been revisited. Relevant data on exposure variables related to human – animal interaction, access to safe water and food habits were re-visited and re-examined and their association with STEC prevalence was determined through bivariate analysis. These results have now been incorporated into the manuscript to support interpretation. Discussion section has been revised to align with the analyzed data).

3. The definition of Shiga toxin producing Escherichia coli O157:H7 relies on the presence of stx1 and/or stx2 genes. However, several isolates (N014, N069, N045, N050, N079, N115, N032, N010B, N006B, N014B) reported in Table

“ANTIBITIC_SUSCEPTITY_DATA_PLOS ONE_SUBMISSION” appear to be positive for the rfbE gene but negative for both stx1 and stx2. Could the authors clarify whether these isolates were still classified as STEC and how this was considered in the prevalence calculation?

Response

(Thank you for this comment, we⁠ have clarified and revised the manuscript accordingly. Specifically, the prevalence of STEC was re-evaluated based⁠ on the presence of stx1 and/or stx2 genes and now is reported as⁠ 13.3%, while the prevalence⁠ of E. coli O157:H7 serotypes remains 20%. The antibiotic susceptibility data were reanalyzed based on 13% confirmed STEC isolates).

4. The table “ANTIBITIC_SUSCEPTITY_DATA_PLOS_ONE_SUBMISSION” includes both virulence gene detection and antimicrobial susceptibility data. For clarity, the authors may consider either revising the table title to reflect both datasets or separating the information into two tables.

Response

(The table title has been revised accordingly to clearly reflect the association between virulence genes detection and antimicrobial susceptibility patterns in STEC positive isolates)

5. In addition, this dataset is currently provided in the supplementary material. It would improve readability if a summarized table of virulence gene prevalence and antimicrobial susceptibility were included in the main manuscript

Response

(A summarized table (Table 3) of virulence gene prevalence and antimicrobial susceptibility patterns has been included in the revised manuscript)

6. In Figure 2, the correspondence between gel lanes and the Sample IDs listed in Table “ANTIBITIC_SUSCEPTITY_DATA_PLOS_ONE_SUBMISSION” is not indicated. The authors should clarify which isolates

correspond to each lane.

Response

(Gel wells were labeled numerically due to limited space on the gel doc. The figure just provided a representative

image from multiple PCR runs, so not all isolates appeared in the same gel. The correspondence between lane numbers and sample IDs (e.g., lane 7 = N011B, lane 11 = N003BZL, lane 15 = N021) has now been clarified in the figure legend).

7. In addition, the expected band sizes for each virulence gene should be annotated in the gel, and the image quality (resolution/contrast) could be improved to facilitate interpretation of the PCR products.

Response

(The expected band sizes for each virulence gene have now been clearly indicated in the figure and described in the legend to facilitate interpretation. While we acknowledge the limitation⁠ in image resolution due to the original gel

documentation,⁠ we have optimized the contrast as much as possible to improve visibility).

8. Remove STEC in the tittle Line 68 = change “Shiga toxin-producing E. coli STEC” by only “STEC” Line 239 = change “aea” to “eae” Line 323 = change “Shiga toxin producing E.coli” to “STEC” Line 334 = change “aea” to “eae” Line 334 = “…hemolysin gene EHEC hly was identified…” this gene is the hlyA? describe the full name of the hemolysin gene.

Response

(All terminology has been standardized by replacing redundant phrase with “STEC” and correcting typographical errors (e.g., “aea” to “ea⁠e”). The hemolysin gene has been clarified as hlyA and fully described as enterohemorrhagic Escherichia coli hemolysin A (EHEC hlyA). All changes have been incorporated into

the revised⁠ manuscript).

COMMENTS FROM REVIEWER 2

1. While the study is interesting and the frequency of STEC O157:H7 (20%) is striking, the article requires a more in-depth analysis of the collected data. A more thorough analysis of the sociodemographic and clinical information in relation to the isolates obtained is lacking. In all cases, only percentage values are mentioned, without any correlation analysis. It is noteworthy that, given the high frequency of STEC, these patients were not followed up. Another weakness of the manuscript is the presentation of the results, which presents raw data without adequate statistical analysis. The manuscript provides too much information, not necessarily related, especially in the introduction. The discussion is weak, and given the lack of statistical analysis, it is not possible to draw clear conclusions.

Response

(We thank the reviewer for the comments. In-depth statistical analysis has been conducted using Firth logistic regression. Correlation between STEC prevalence and exposure factors (human–animal contact, dietary habits, and access to clean and safe water) has been analyzed and included. Results section has been improved to include statistical outputs beyond

percentages. Discussion has been strengthened to reflect analytical findings and support

clearer conclusions. Introduction has been revised to remove irrelevant information and improve focus. Overall manuscript has been revised to enhance clarity, coherence, and scientific rigor)

COMMENTS FROM REVIEWER 3

1. However, there are several methodological, conceptual, and structural limitations that need to be addressed. These include issues related to study design, microbiological characterization, and data presentation. In addition, several aspects of the manuscript would benefit from improved clarity, updated references, and better organization. In its current form, the manuscript requires major revision before it can be considered for publication.

Response

(We thank the reviewer for the comments. All concerns have been addressed,⁠ and the manuscript has been revised to improve methodology, data presentation, clarity, organization and references)

Attachment Submitted filename: Letter_response_to_reviewers.pdf

# Peer review: Decision Letter 1

**DOI:** 10.1371/journal.pone.0353396.r003

**Authors:** Fernando Navarro-Garcia, Fernando Navarro-Garcia

16 Jun 2026

PONE-D-26-07244R1

Prevalence, virulence profiles and antibiotic susceptibility patterns of Shiga toxin producing Escherichia coli O157:H7 among children 6-59 months in Longido, Arusha-Tanzania.

PLOS One

Dear Dr. Martin,

Thank you for submitting your manuscript to PLOS ONE. After careful consideration, we feel that it has merit but does not fully meet PLOS ONE’s publication criteria as it currently stands. Therefore, we invite you to submit a revised version of the manuscript that addresses the points raised during the review process.

Please submit your revised manuscript by Jul 31 2026 11:59PM. If you will need more time than this to complete your revisions, please reply to this message or contact the journal office at plosone@plos.org. When you're ready to submit your revision, log on to https://www.editorialmanager.com/pone/ and select the 'Submissions Needing Revision' folder to locate your manuscript file.

Please include the following items when submitting your revised manuscript:

- A letter that responds to each point raised by the academic editor and reviewer(s). You should upload this letter as a separate file labeled 'Response to Reviewers'.
- A marked-up copy of your manuscript that highlights changes made to the original version. You should upload this as a separate file labeled 'Revised Manuscript with Track Changes'.
- An unmarked version of your revised paper without tracked changes. You should upload this as a separate file labeled 'Manuscript'.

If you would like to make changes to your financial disclosure, please include your updated statement in your cover letter. Guidelines for resubmitting your figure files are available below the reviewer comments at the end of this letter.

If applicable, we recommend that you deposit your laboratory protocols in protocols.io to enhance the reproducibility of your results. Protocols.io assigns your protocol its own identifier (DOI) so that it can be cited independently in the future. For instructions see: https://journals.plos.org/plosone/s/submission-guidelines#loc-laboratory-protocols. Additionally, PLOS ONE offers an option for publishing peer-reviewed Lab Protocol articles, which describe protocols hosted on protocols.io. Read more information on sharing protocols at https://plos.org/protocols?utm_medium=editorial-email&utm_source=authorletters&utm_campaign=protocols.

As the corresponding author, your ORCID iD is verified in the submission system and will appear in the published article. PLOS supports the use of ORCID, and we encourage all coauthors to register for an ORCID iD and use it as well. Please encourage your coauthors to verify their ORCID iD within the submission system before final acceptance, as unverified ORCID iDs will not appear in the published article. *Only*  the individual author can complete the verification step; PLOS staff *cannot*  verify ORCID iDs on behalf of authors.

We look forward to receiving your revised manuscript.

Kind regards,

Fernando Navarro-Garcia

Academic Editor

PLOS One

**Journal Requirements:**

1. If the reviewer comments include a recommendation to cite specific previously published works, please review and evaluate these publications to determine whether they are relevant and should be cited. There is no requirement to cite these works unless the editor has indicated otherwise.

2. Please review your reference list to ensure that it is complete and correct. If you have cited papers that have been retracted, please include the rationale for doing so in the manuscript text, or remove these references and replace them with relevant current references. Any changes to the reference list should be mentioned in the rebuttal letter that accompanies your revised manuscript. If you need to cite a retracted article, indicate the article’s retracted status in the References list and also include a citation and full reference for the retraction notice.

[Note: HTML markup is below. Please do not edit.]

Reviewers' comments:

Reviewer's Responses to Questions

**Comments to the Author**

1. If the authors have adequately addressed your comments raised in a previous round of review and you feel that this manuscript is now acceptable for publication, you may indicate that here to bypass the “Comments to the Author” section, enter your conflict of interest statement in the “Confidential to Editor” section, and submit your "Accept" recommendation.

Reviewer #1: All comments have been addressed

Reviewer #3: (No Response)

**********

2. Is the manuscript technically sound, and do the data support the conclusions?

The manuscript must describe a technically sound piece of scientific research with data that supports the conclusions. Experiments must have been conducted rigorously, with appropriate controls, replication, and sample sizes. The conclusions must be drawn appropriately based on the data presented.

Reviewer #1: Yes

Reviewer #3: Yes

**********

3. Has the statistical analysis been performed appropriately and rigorously?

Reviewer #1: Yes

Reviewer #3: Yes

**********

4. Have the authors made all data underlying the findings in their manuscript fully available?

The PLOS Data policy requires authors to make all data underlying the findings described in their manuscript fully available without restriction, with rare exception (please refer to the Data Availability Statement in the manuscript PDF file). The data should be provided as part of the manuscript or its supporting information, or deposited to a public repository. For example, in addition to summary statistics, the data points behind means, medians and variance measures should be available. If there are restrictions on publicly sharing data—e.g. participant privacy or use of data from a third party—those must be specified.

Reviewer #1: Yes

Reviewer #3: Yes

**********

5. Is the manuscript presented in an intelligible fashion and written in standard English?

PLOS ONE does not copyedit accepted manuscripts, so the language in submitted articles must be clear, correct, and unambiguous. Any typographical or grammatical errors should be corrected at revision, so please note any specific errors here.

Reviewer #1: Yes

Reviewer #3: No

**********

6. Review Comments to the Author

Please use the space provided to explain your answers to the questions above. You may also include additional comments for the author, including concerns about dual publication, research ethics, or publication ethics. (Please upload your review as an attachment if it exceeds 20,000 characters)

**Reviewer #1:**  (No Response)

**Reviewer #3:**  General Comment

My primary concern is that the revised manuscript does not correspond to the tracked-changes version provided by the authors. The two documents differ substantially, making the review process unnecessarily difficult, as it essentially required reviewing two different manuscripts. The authors should ensure that the correct tracked-changes file is submitted so that reviewers can accurately assess the revisions made in response to previous comments.

Introduction

The statement "It is estimated to cause millions of infections annually, with a proportion progressing to severe disease requiring hospitalization" lacks quantitative support. How many millions of infections are being referred to? In the original version, the authors cited data from 2014, whereas the tracked-changes version included data from 2020. In the revised manuscript, these data have been removed. The authors should provide up-to-date epidemiological estimates and appropriate references to support this statement.

Regarding the tracked-changes version, the authors improved the Introduction by incorporating more recent references and providing a clearer epidemiological context for STEC in Africa. However, several paragraphs still require language editing, and some grammatical and stylistic issues remain. For example, in the paragraph spanning lines 79–86, the term “Tanzania” is repeated three times unnecessarily.

Additionally, in line 137, there is a repeated phrase “The results of this study provided The findings of this study”

The study objective has been reformulated and now adequately reflects the scope of the results presented.

Methodology

The authors have still not clarified the criteria used to define multidrug resistance (MDR). Specifically, it remains unclear whether MDR was defined as resistance to more than two antimicrobial agents, resistance to three or more antimicrobial classes, or according to another established criterion. A clear definition should be provided.

The tracked-changes version also contains several typographical and grammatical errors. For example:

• Line 187: “Table one”

• Line 188: “Table two”

These should be revised according to the journal style.

In line 202, a punctuation error is present: "Bauer disk diffusion technique (Sangeda et al., 2021) According to the guidelines..." A period is missing after the citation. I recommend that the authors carefully proofread the entire manuscript to identify and correct similar issues.

I also note that the revised manuscript includes an Ethical Clearance section, whereas this information is absent from the tracked-changes version. This discrepancy further highlights the inconsistency between the two submitted documents.

Results

The Results section is presented quite differently in the revised manuscript and in the tracked-changes version. The revised manuscript is more consistent with the recommendations provided during the first round of review and appears to better address the concerns previously raised.

In line 218, the authors refer to “STEC status.” What do they mean by STEC Status?

Overall Assessment

Overall, the authors have addressed several of the comments raised during the previous review round. However, it remains difficult to accurately assess the revisions because the tracked-changes document does not correspond to the revised manuscript currently under review.

The remaining minor comments on the revised version should be addressed. A final round of revision appears necessary, and I strongly recommend that the authors submit the correct tracked-changes version so that reviewers can properly evaluate the modifications made.

**********

7. PLOS authors have the option to publish the peer review history of their article (what does this mean?). If published, this will include your full peer review and any attached files.

If you choose “no”, your identity will remain anonymous but your review may still be made public.

**Do you want your identity to be public for this peer review?**  For information about this choice, including consent withdrawal, please see our Privacy Policy.

Reviewer #1: No

Reviewer #3: No

**********

[NOTE: If reviewer comments were submitted as an attachment file, they will be attached to this email and accessible via the submission site. Please log into your account, locate the manuscript record, and check for the action link "View Attachments". If this link does not appear, there are no attachment files.]

To ensure your figures meet our technical requirements, please review our figure guidelines: https://journals.plos.org/plosone/s/figures

You may also use PLOS’s free figure tool, NAAS, to help you prepare publication quality figures: https://journals.plos.org/plosone/s/figures#loc-tools-for-figure-preparation.

NAAS will assess whether your figures meet our technical requirements by comparing each figure against our figure specifications.

# Author comment: Author response to Decision Letter 2

**DOI:** 10.1371/journal.pone.0353396.r004

19 Jun 2026

Reviewer #3 comments

1. The statement "It is estimated to cause millions of infections annually, with a proportion progressing to severe disease requiring hospitalization" lacks quantitative support. How many millions of infections are being referred to?

Response

Thank you for this comment. We have revised the statement to provide a specific quantitative estimate. The text

now reads⁠: "It is estimated to cause 2.8 million acute illnesses annually. The corresponding reference has been retained to support this estimate

2. The authors should provide up-to-date epidemiological estimates and appropriate references to support this statement.

Response

Thank you for this valuable comment. We have updated the epidemiological information by replacing the older estimates with recent data of 2020.

3. Several paragraphs still require language editing, and some grammatical and stylistic issues remain. For example, in the paragraph spanning lines 79–86, the term “Tanzania” is repeated three times unnecessarily. Additionally, in line 137, there is a repeated phrase “The results of this study provided The findings of this study”

Response

Thank you for your careful review and constructive comments. The unnecessary repetition of the term “

Tanzania” in lines 79–86 has been corrected and the duplicated phrase in⁠ line 137 has been⁠ removed.

4. The authors have still not clarified the criteria used to define multidrug resistance (MDR). Specifically, it remains unclear whether MDR was defined as resistance to more than two antimicrobial agents, resistance to three or more antimicrobial classes, or according to another established criterion. A clear definition should be provided.

Response

Thank you for this important comment. We have clarified the definition of multidrug resistance (MDR) in the revised manuscript. MDR is now explicitly defined as acquired resistance to at least one antimicrobial agent in three or more antimicrobial classes, in line⁠ with standard international criteria.

5. The tracked-changes version also contains several typographical and grammatical errors. For example:

• Line 187: “Table one”

• Line 188: “Table two”

Response

These has been revised according to the journal requirements.

6.In line 202, a punctuation error is present: "Bauer disk diffusion technique (Sangeda et al., 2021) According to the guidelines..." A period is missing after the citation. I recommend that the authors carefully proofread the entire manuscript to identify and correct similar issues.

Response

Thank you for identifying this error. We have corrected the punctuation by inserting a full stop⁠ after the citation, so that the sentence now reads appropriately

7. I also note that the revised manuscript includes an Ethical Clearance section, whereas this information is absent from the tracked-changes version. This discrepancy further highlights the inconsistency between the two submitted

documents.

Response

Thank you for this observation.⁠ We acknowledge the inconsistency between the previously submitted tracked-changes

and clean version. The tracked-changes manuscript has now been updated to fully match the revised clean manuscript. Both documents have been carefully checked to ensure consistency and completeness.

8.In line 218, the authors refer to “STEC status.” What do they mean by STEC Status?

Response

Thank you for this comment. We acknowledge that the term⁠ “STEC status” was unclear in the original manuscript.

This has been corrected in the revised version by using a clear and more precise description

Attachment Submitted filename: Response to Reviewers.pdf

# Peer review: Decision Letter 2

**DOI:** 10.1371/journal.pone.0353396.r005

**Authors:** Fernando Navarro-Garcia, Fernando Navarro-Garcia, Fernando Navarro-Garcia

24 Jun 2026

Prevalence, virulence profiles and antibiotic susceptibility patterns of Shiga toxin producing Escherichia coli O157:H7 among children 6-59 months in Longido, Arusha-Tanzania.

PONE-D-26-07244R2

Dear Dr. Martin,

We’re pleased to inform you that your manuscript has been judged scientifically suitable for publication and will be formally accepted for publication once it meets all outstanding technical requirements.

Within one week, you’ll receive an e-mail detailing the required amendments. When these have been addressed, you’ll receive a formal acceptance letter and your manuscript will be scheduled for publication.

An invoice will be generated when your article is formally accepted. Please note, if your institution has a publishing partnership with PLOS and your article meets the relevant criteria, all or part of your publication costs will be covered. Please make sure your user information is up-to-date by logging into Editorial Manager at Editorial Manager® and clicking the ‘Update My Information' link at the top of the page. For questions related to billing, please contact billing support.

If your institution or institutions have a press office, please notify them about your upcoming paper to help maximize its impact. If they’ll be preparing press materials, please inform our press team as soon as possible -- no later than 48 hours after receiving the formal acceptance. Your manuscript will remain under strict press embargo until 2 pm Eastern Time on the date of publication. For more information, please contact onepress@plos.org.

Kind regards,

Fernando Navarro-Garcia

Academic Editor

PLOS One

Additional Editor Comments (optional):

Reviewers' comments:

Reviewer's Responses to Questions

**Comments to the Author**

1. If the authors have adequately addressed your comments raised in a previous round of review and you feel that this manuscript is now acceptable for publication, you may indicate that here to bypass the “Comments to the Author” section, enter your conflict of interest statement in the “Confidential to Editor” section, and submit your "Accept" recommendation.

Reviewer #3: (No Response)

**********

2. Is the manuscript technically sound, and do the data support the conclusions?

The manuscript must describe a technically sound piece of scientific research with data that supports the conclusions. Experiments must have been conducted rigorously, with appropriate controls, replication, and sample sizes. The conclusions must be drawn appropriately based on the data presented.

Reviewer #3: Yes

**********

3. Has the statistical analysis been performed appropriately and rigorously?

Reviewer #3: Yes

**********

4. Have the authors made all data underlying the findings in their manuscript fully available?

The PLOS Data policy requires authors to make all data underlying the findings described in their manuscript fully available without restriction, with rare exception (please refer to the Data Availability Statement in the manuscript PDF file). The data should be provided as part of the manuscript or its supporting information, or deposited to a public repository. For example, in addition to summary statistics, the data points behind means, medians and variance measures should be available. If there are restrictions on publicly sharing data—e.g. participant privacy or use of data from a third party—those must be specified.

Reviewer #3: Yes

**********

5. Is the manuscript presented in an intelligible fashion and written in standard English?

PLOS ONE does not copyedit accepted manuscripts, so the language in submitted articles must be clear, correct, and unambiguous. Any typographical or grammatical errors should be corrected at revision, so please note any specific errors here.

Reviewer #3: Yes

**********

6. Review Comments to the Author

Please use the space provided to explain your answers to the questions above. You may also include additional comments for the author, including concerns about dual publication, research ethics, or publication ethics. (Please upload your review as an attachment if it exceeds 20,000 characters)

Reviewer #3: The authors have addressed all comments raised by this reviewer, except for clarifying what they mean by "STEC status."

I have no further comments.

**********

7. PLOS authors have the option to publish the peer review history of their article (what does this mean?). If published, this will include your full peer review and any attached files.

If you choose “no”, your identity will remain anonymous but your review may still be made public.

**Do you want your identity to be public for this peer review?**  For information about this choice, including consent withdrawal, please see our Privacy Policy.

Reviewer #3: No

**********

# Editor report: Acceptance letter

**DOI:** 10.1371/journal.pone.0353396.r006

**Authors:** Fernando Navarro-Garcia, Fernando Navarro-Garcia, Fernando Navarro-Garcia

PONE-D-26-07244R2

PLOS One

Dear Dr. Martin,

I'm pleased to inform you that your manuscript has been deemed suitable for publication in PLOS One. Congratulations! Your manuscript is now being handed over to our production team.

At this stage, our production department will prepare your paper for publication. This includes ensuring the following:

* All references, tables, and figures are properly cited

* All relevant supporting information is included in the manuscript submission,

* There are no issues that prevent the paper from being properly typeset

You will receive further instructions from the production team, including instructions on how to review your proof when it is ready. Please keep in mind that we are working through a large volume of accepted articles, so please give us a few days to review your paper and let you know the next and final steps.

Lastly, if your institution or institutions have a press office, please let them know about your upcoming paper now to help maximize its impact. If they'll be preparing press materials, please inform our press team within the next 48 hours. Your manuscript will remain under strict press embargo until 2 pm Eastern Time on the date of publication. For more information, please contact onepress@plos.org.

You will receive an invoice from PLOS for your publication fee after your manuscript has reached the completed accept phase. If you receive an email requesting payment before acceptance or for any other service, this may be a phishing scheme. Learn how to identify phishing emails and protect your accounts at https://explore.plos.org/phishing.

If we can help with anything else, please email us at customercare@plos.org.

Thank you for submitting your work to PLOS ONE and supporting open access.

Kind regards,

PLOS ONE Editorial Office Staff

on behalf of

Dr. Fernando Navarro-Garcia

Academic Editor

PLOS One
